# ANXA2 as a Candidate Molecular Hub Linking Fibrinolysis, Coagulation, and Immune Remodeling in Hepatocellular Carcinoma: An Integrative Multi-Omics Analysis

**DOI:** 10.3390/ijms27198915

**Published:** 2026-10-07

**Authors:** Esra’a Abudouleh, Rasha Elsayim, Heba W. Alhamdi, Nada Fahad Almebairik, Noorah A. Alkubaisi

**Affiliations:** 1 Department of Botany and Microbiology, College of Science, King Saud University, P.O. Box 2455, Riyadh 11451, Saudi Arabia; 2 Department of Biology, College of Sciences, King Khalid University, P.O. Box 960, Abha 61413, Saudi Arabia; 3 Department of Clinical Laboratory Science, College of Applied Medical Sciences, King Saud University, P.O. Box 10219, Riyadh 11433, Saudi Arabia

**Keywords:** hepatocellular carcinoma, *ANXA2*, inflammation, coagulation, fibrinolysis, bioinformatics

## Abstract

Annexin A2 (ANXA2) is involved in cell-surface fibrinolysis and has been associated with hepatocellular carcinoma (HCC). We examined *ANXA2* expression in HCC and its associations with survival and immune–hemostatic gene expression. *ANXA2* transcript expression was assessed in The Cancer Genome Atlas liver cancer cohort (TCGA-LIHC) and an independent HCC dataset (GSE25097). Public protein datasets were used to examine *ANXA2* abundance. We analyzed clinicopathological associations, overall survival, tumor–normal tissue discrimination, and correlations with selected immune, coagulation, fibrinolytic, and inflammatory genes. *ANXA2* expression was higher in HCC than in non-tumor liver tissue in the transcriptomic analyses and was elevated in the protein analyses. Higher *ANXA2* expression was associated with poorer overall survival after adjustment for age, sex, and pathological stage (hazard ratio, 1.26; 95% confidence interval, 1.07–1.49; *p* = 0.006). Within TCGA-LIHC, *ANXA2* expression discriminated tumor from normal tissue (area under the curve, 0.895; 95% confidence interval, 0.852–0.933); this performance was not independently validated. *ANXA2* expression also correlated with selected fibrinolytic, inflammatory, and immune-related genes. GSE25097 supported the differential-expression finding. *ANXA2* is overexpressed in HCC and is associated with poorer survival and an immune–hemostatic transcriptional pattern. The correlations do not establish that *ANXA2* regulates these processes.

## 1. Introduction

Hepatocellular carcinoma (HCC) is the most common type of primary liver cancer and a major cause of cancer-related mortality [1,2,3,4]. Although significant progress has been made in the molecular classification and development of targeted therapies for HCC, patient prognosis remains unfavorable. This is largely attributed to the challenges of late-stage diagnosis, frequent tumor recurrence, and pronounced heterogeneity within tumors. Emerging evidence suggests that HCC progression is not solely a result of tumor-cell alterations. Instead, it is significantly affected by the complex interactions between inflammatory signaling pathways, immune system responses, and the remodeling of stromal tissues [5,6,7]. Understanding the interconnected molecular networks within the tumor microenvironment may yield more comprehensive insights into the progression of HCC than examining individual pathways separately.

Chronic inflammation is a fundamental aspect of hepatocarcinogenesis, particularly in cases involving viral hepatitis, metabolic disorders, and liver fibrosis. The ongoing inflammatory signaling contributes to the initiation and advancement of tumors by influencing cytokine pathways and altering immune cell functions [8,9]. In parallel, the immune microenvironment of HCC is characterized by considerable heterogeneity, encompassing immune cell populations and signaling pathways that can either promote tumor surveillance or facilitate tumor progression and immune evasion [3,10]. The diversity present also makes it challenging to identify biomarkers that indicate how advanced HCC will respond to treatments targeting PD-1/PD-L1. Zhang et al. examined potential biomarkers, such as the expression of PD-L1 in tumors and characteristics of the tumor’s immune microenvironment, emphasizing the importance of considering differences among patients and tumors [11]. These insights underscore the necessity of viewing inflammatory and immune processes as interconnected elements within the HCC tumor microenvironment, rather than as distinct mechanisms.

Alongside changes in inflammatory and immune responses, the hemostatic system has become a significant aspect of HCC biology. Disruptions in coagulation and fibrinolysis are commonly observed in HCC, and these disruptions are linked to tumor growth, metastasis, and unfavorable clinical outcomes [6,12]. Tumor-induced hypercoagulability, such as portal vein thrombosis, not only results from advanced disease but may also promote tumor progression by influencing angiogenesis, extracellular matrix remodeling, and immune cell activity [13,14,15]. Moreover, there is increasing evidence that coagulation and fibrinolytic pathways interact with inflammatory and immune signaling within the tumor microenvironment, establishing a biological link between hemostasis and tumor-associated inflammation.

The concept of immunothrombosis describes the convergence of immune and hemostatic pathways, where immune cells, platelets, coagulation factors, and fibrinolytic components interact within the tissue microenvironment [6,9]. In cancer, these interactions can impact thrombosis, inflammatory signaling, vascular function, extracellular matrix remodeling, and tumor progression. Although immunothrombotic mechanisms have been thoroughly explored in inflammatory and vascular disorders, the molecular factors that coordinate these processes in HCC are not well understood. Therefore, identifying molecules that link immune and hemostatic pathways could provide valuable insights into the biological mechanisms driving HCC progression.

Annexin A2 (ANXA2) is a protein that has garnered attention for its potential role in bridging hemostatic and immune functions. As a calcium-dependent phospholipid-binding protein, *ANXA2* is integral to cell-surface fibrinolysis, enhancing the interaction between tissue plasminogen activator and plasminogen, which in turn promotes plasmin generation and fibrinolytic activity [6,12,16,17,18,19]. Beyond its role in fibrinolysis, *ANXA2* is involved in extracellular matrix remodeling, tumor invasion, inflammatory signaling, and immune regulation [8,20,21]. These multifaceted roles suggest that *ANXA2* may act as a molecular conduit linking fibrinolysis, inflammation, and the tumor immune microenvironment.

*ANXA2* overexpression in HCC has been correlated with tumor advancement, unfavorable prognosis, and resistance to treatment [7]. Recent investigations have proposed a link between *ANXA2* levels and the immune microenvironment, particularly in mechanisms of immune evasion [3,10]. Despite this, most research has concentrated on *ANXA2*’s role in isolated facets of HCC biology, neglecting its potential involvement in a comprehensive immune–hemostatic network. Notably, the interplay between *ANXA2* and the synchronized regulation of coagulation, fibrinolysis, inflammatory pathways, and immune remodeling in HCC is not yet thoroughly understood.

Earlier research has explored the expression of *ANXA2*, its prognostic implications, and its connections to the immune system in HCC. This study builds on that foundation by investigating the relationship between *ANXA2* expression and transcriptional patterns related to fibrinolysis, coagulation, inflammation, and immunity in HCC. We evaluated *ANXA2* expression through transcriptomic and protein datasets, explored its links to clinicopathological characteristics and overall survival, and examined genes related to immunity and hemostasis, as well as enriched pathways. Additionally, *ANXA2* differential expression was analyzed in a separate HCC transcriptomic dataset. Collectively, these analyses outline an immune–hemostatic pattern associated with *ANXA2* that merits further exploration; they do not confirm that *ANXA2* governs these processes.

## 2. Results

### 2.1. ANXA2 Is Overexpressed in LIHC

According to the Tumor Immune Estimation Resource (TIMER) analysis of The Cancer Genome Atlas Liver Hepatocellular Carcinoma (TCGA-LIHC), ANXA2 transcript levels were significantly higher in HCC tissue compared to normal liver tissue (*p* < 0.001; Figure S1A). Similarly, Gene Expression Profiling Interactive Analysis (GEPIA) demonstrated increased expression in HCC using TCGA tumor data and TCGA/GTEx normal tissue data (*p* < 0.01; Figure S1B). Since both analyses involve TCGA-LIHC tumors, they offer complementary insights into overlapping data rather than serving as independent validation.

### 2.2. ANXA2 Expression Across Clinicopathological Subgroups

**Figure 1:**
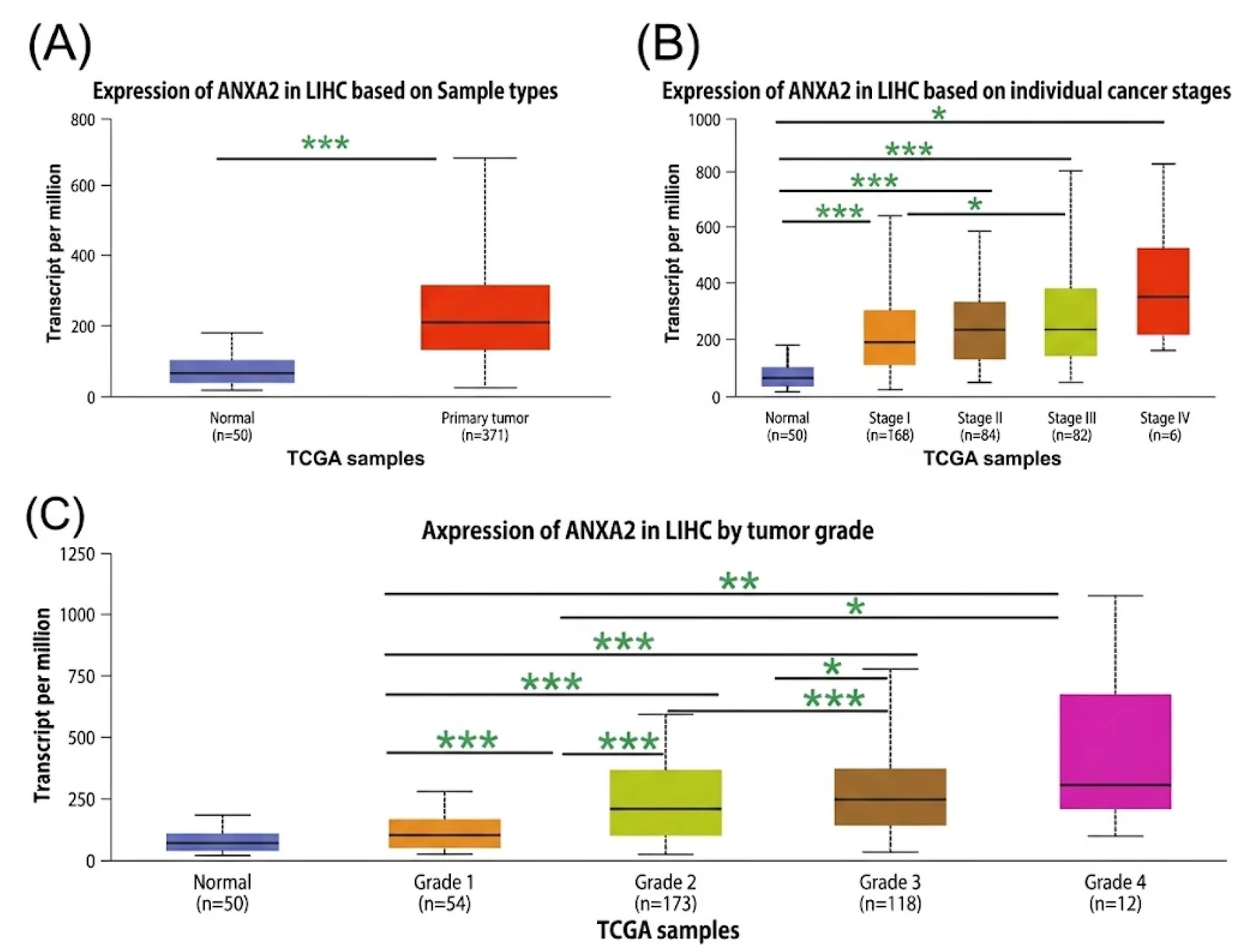
Association of *ANXA2* expression with clinicopathological features. (**A**) Comparison of *ANXA2* levels between normal liver tissues (*n* = 50) and primary LIHC tissues (*n* = 371). (**B**) *ANXA2* expression by pathological stage: stage I (*n* = 168), stage II (*n* = 84), stage III (*n* = 82), and stage IV (*n* = 6). (**C**) *ANXA2* expression across tumor grades: grade 1 (*n* = 54), grade 2 (*n* = 173), grade 3 (*n* = 118), and grade 4 (*n* = 12). The expression levels are quantified as transcripts per million (TPM). Statistical analyses were executed using the standard procedures in UALCAN, with horizontal lines indicating the groups compared. Statistical significance is indicated as follows: *p* < 0.01 (*), *p* < 0.001 (**), *p* < 0.0001 (***). The central line within each box indicates the median, and the vertical dashed lines represent the boxplot whiskers. Horizontal black lines above the groups indicate the statistical comparisons.

In the University of ALabama at Birmingham CANcer data analysis Portal.

(UALCAN), the expression of *ANXA2* was found to be significantly elevated in primary HCC tumors (*n* = 371) compared to normal liver samples (*n* = 50; *p* < 0.001; Figure 1A). For stages I–III, the expression levels were notably higher than those in normal tissue (all *p* < 0.001), while the comparison for stage IV showed a *p*-value of less than 0.05 (Figure 1B). Furthermore, *ANXA2* expression was consistently higher across different tumor-grade subgroups when compared to normal tissue (Figure 1C). However, the small sample sizes of stage IV (*n* = 6) and grade 4 (*n* = 12) groups pose limitations on interpreting differences among tumor subgroups.

### 2.3. Elevated ANXA2 Protein Abundance in HCC

**Figure 2:**
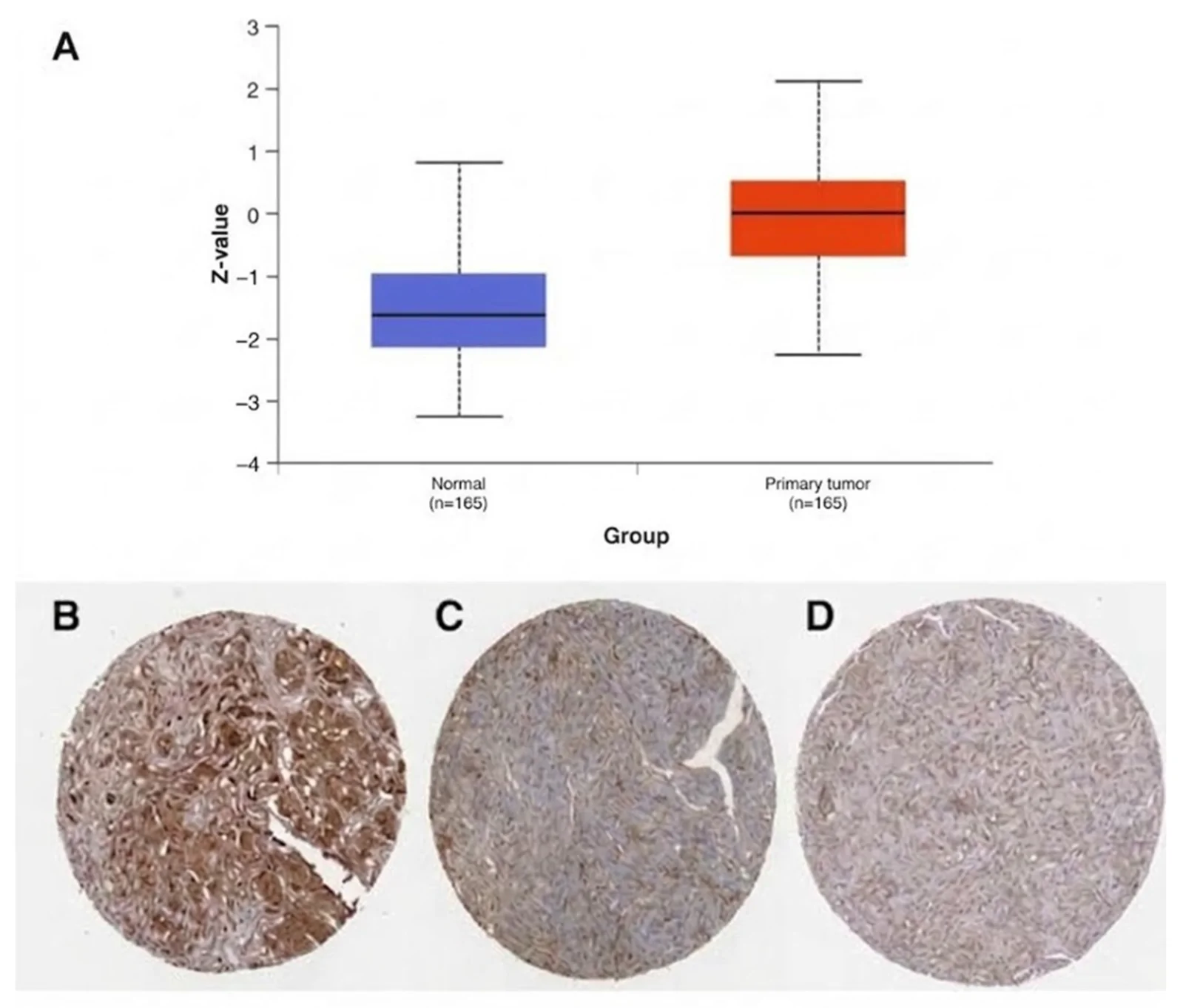
Protein level expression of ANXA2. (**A**) Data from the UALCAN platform, sourced from CPTAC, reveal ANXA2 protein levels in normal liver tissues (*n* = 165) and primary LIHC tissues (*n* = 165). These levels are expressed as Z-score-normalized values. The analysis indicates a significantly elevated abundance of *ANXA2* protein in primary tumor tissues compared to normal tissues (unpaired two-sample *t*-test, *p* = 5.31 × 10^−31^) The central line within each box indicates the median, and the vertical dashed lines represent the boxplot whiskers. The horizontal black line above the groups indicates the statistical comparison. (**B**–**D**) Immunohistochemical images from the Human Protein Atlas demonstrate varied *ANXA2* staining patterns in hepatocellular carcinoma tissues, with localization primarily in the cytoplasm and membrane.

In the Clinical Proteomic Tumor Analysis Consortium (CPTAC) analysis, the abundance of *ANXA2* protein was significantly greater in HCC compared to normal liver tissue, with both groups comprising 165 samples (*p* = 5.31 × 10^−31^; Figure 2A). Examination of Human Protein Atlas images indicated varying levels of *ANXA2* staining in the HCC samples, predominantly observed in the cytoplasm and membrane (Figure 2B–D).

### 2.4. External Transcriptomic Validation

*ANXA2* expression was analyzed using the independent GEO dataset GSE25097, which consists of 268 HCC tissues and 243 adjacent non-tumor liver tissues. The analysis of differential expression demonstrated a notable increase in *ANXA2* levels within HCC tissues, adhering to the criteria of log_2_FC ≥ 0.585 and an adjusted *p*-value of less than 0.01 (Figure S2). This independent cohort supports the consistency of elevated *ANXA2* transcript levels in HCC.

### 2.5. Higher ANXA2 Expression Is Associated with Poorer Overall Survival

**Figure 3:**
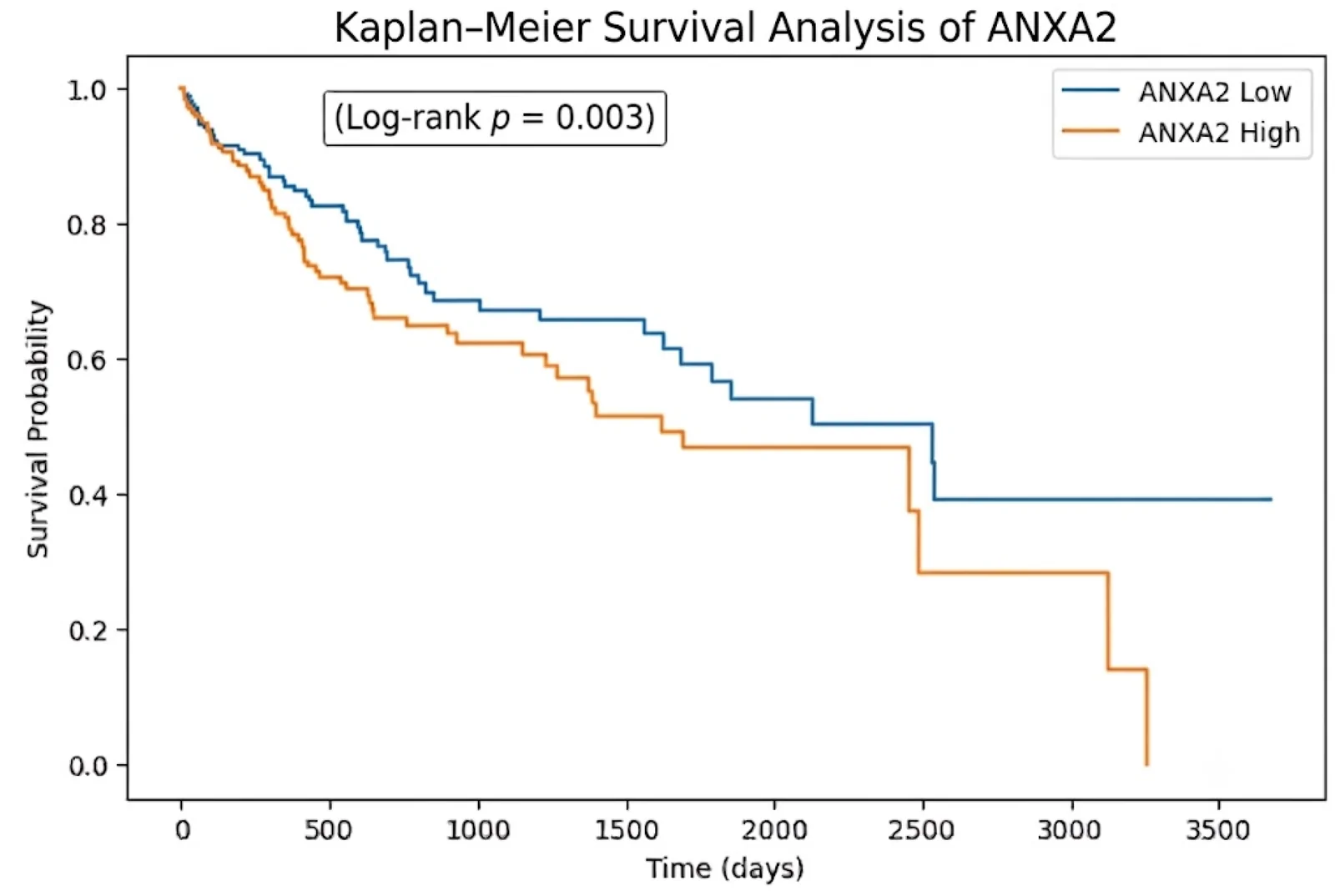
Association of *ANXA2* expression with overall survival in TCGA-LIHC. Kaplan–Meier survival curves are used to compare patients with high (orange) and low (blue) levels of *ANXA2* expression. The study analyzed data from 337 patients, all of whom had complete records on expression, survival, and clinicopathological characteristics, with 114 deaths documented. Patients were categorized into high- and low-expression groups based on the cutoff defined in Section 4.5. The group with elevated *ANXA2* expression demonstrated significantly reduced overall survival compared to the group with lower expression (log-rank *p* = 0.003). Survival time is reported in days.

The link between *ANXA2* expression levels and overall survival was investigated in a cohort of 337 TCGA-LIHC patients, all of whom had complete data on expression, survival, and clinicopathological characteristics, including 114 deaths. Kaplan–Meier analysis indicated that patients with elevated *ANXA2* expression experienced significantly worse overall survival compared to those with lower expression levels (log-rank *p* = 0.003; Figure 3). When considering *ANXA2* expression as a continuous variable, univariate Cox proportional hazards regression revealed that increased expression was linked to a higher risk of death (hazard ratio (HR) = 1.29, 95% confidence intervals (CIs): 1.09–1.53, *p* = 0.003; Table 1). In a multivariable model that accounted for age, sex, and pathological stage, *ANXA2* expression continued to be significantly associated with overall survival (adjusted HR = 1.26, 95% CI: 1.07–1.49, *p* = 0.006). Thus, each one-unit increase in log_2_-transformed *ANXA2* expression corresponded to an estimated 26% rise in the hazard of death, after adjusting for the covariates.

Furthermore, advanced pathological stages (III–IV) were significantly correlated with a higher hazard of death compared to early stages (I–II; adjusted HR = 2.48, 95% CI: 1.70–3.61, *p* < 0.001). Age and sex did not show statistical significance in the adjusted model. The model’s concordance index was 0.64, reflecting moderate discriminatory ability. Cross-platform survival analyses using the Human Protein Atlas and Kaplan–Meier Plotter also demonstrated significantly poorer overall survival for patients with high *ANXA2* expression (Figure S3). In the Human Protein Atlas (HPA) analysis, high *ANXA2* expression was linked to poorer overall survival (log-rank *p* < 0.001), and the Kaplan–Meier Plotter confirmed these findings (HR = 2.12, 95% CI: 1.49–3.03; log-rank *p* = 1.9 × 10^−5^). These results collectively support the association between elevated *ANXA2* expression and adverse overall survival outcomes in LIHC.

### 2.6. ANXA2 Expression and Immune-Cell Features in HCC

In the TIMER analysis, higher *ANXA2* expression was associated with poorer overall survival (log-rank *p* = 0.005). The estimated abundance of B cells, CD8^+^ T cells, CD4^+^ T cells, macrophages, neutrophils, and dendritic cells was not significantly associated with overall survival in the respective analyses (all log-rank *p* > 0.05; Figure S4).

### 2.7. ANXA2 Expression Correlates with Immune Checkpoint- and Macrophage-Related Genes

In the TCGA-LIHC bulk tissue dataset, *ANXA2* expression was found to be positively associated with various genes linked to immune checkpoints and macrophages, as shown in Table S2. Among these, HAVCR2 and CSF1R exhibited the most significant correlations, each with a coefficient of r = 0.45. Conversely, *LAG3* and *IDO1* did not achieve the *p* < 0.01 significance level required for this analysis. It should be noted that these associations do not confirm the presence of checkpoint activity, increased macrophage infiltration, or macrophage polarization.

### 2.8. Functional Pathways Associated with ANXA2 Expression

The expression profile linked to ANXA2 revealed a positive enrichment of gene sets involved in phagocytosis, immune responses, and translation. In contrast, gene sets associated with liver metabolism and certain coagulation processes exhibited negative enrichment (Figure S5; Table S3 and Table S4). Detailed KEGG and Reactome pathway analyses are provided in Supplementary Table S3 and Table S4, respectively… It should be noted that enrichment signifies an association with the expression profile and does not confirm that ANXA2 directly activates or inhibits these pathways.

### 2.9. Correlations of ANXA2 with Coagulation-, Fibrinolysis-, and Inflammation-Related Genes in LIHC

**Figure 4:**
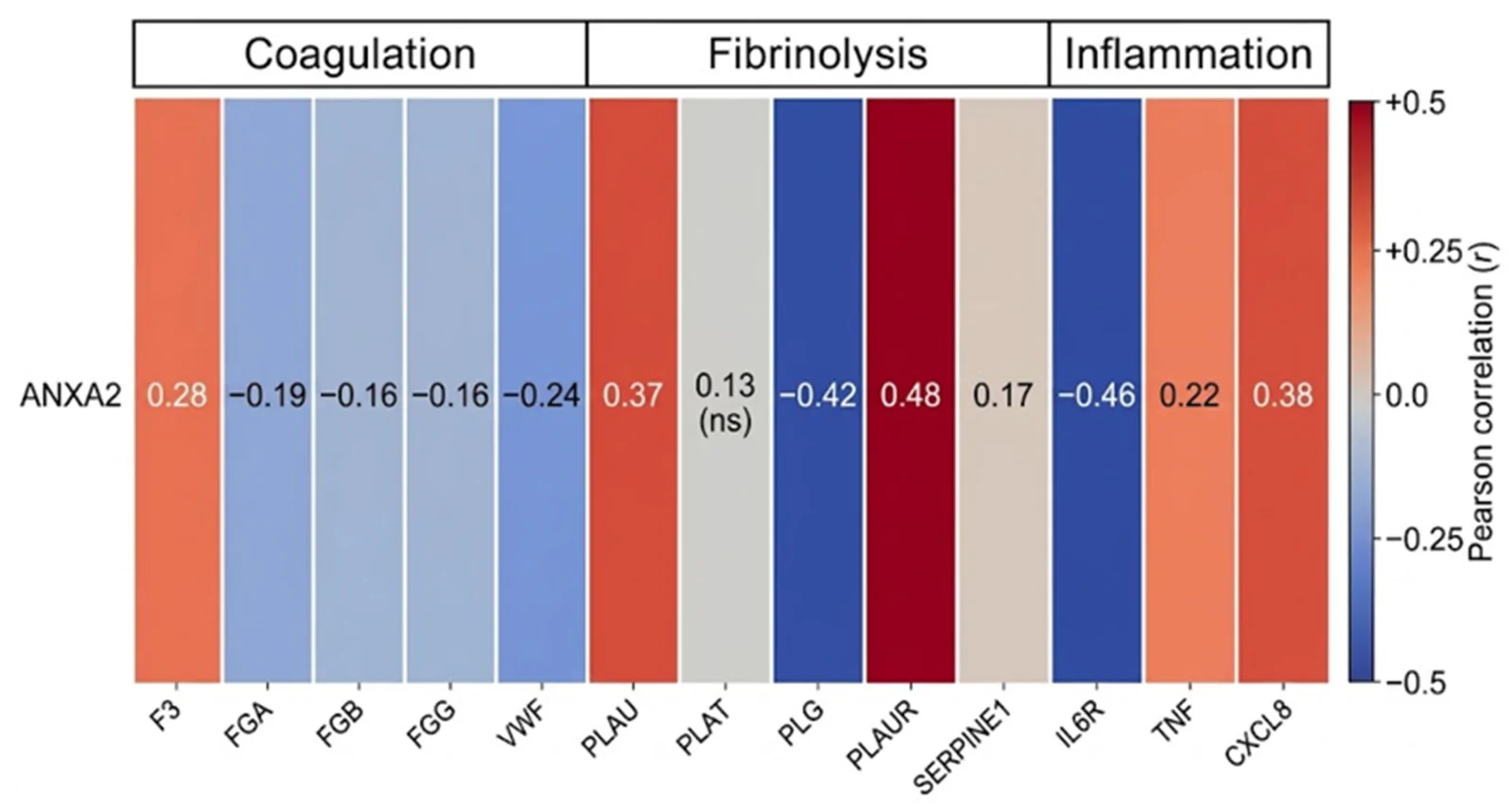
Correlations between *ANXA2* and coagulation-, fibrinolysis-, and inflammation-related gene expression in HCC. The heatmap shows Pearson correlation coefficients calculated from bulk-tissue mRNA expression in the TCGA-LIHC cohort using LinkedOmics. Genes are grouped as coagulation-related (*F3, FGA, FGB, FGG, and VWF*), fibrinolysis-related (*PLAU, PLAT, PLG, PLAUR*, and *SERPINE1*), and inflammation-related (*IL6R, TNF*, and *CXCL8 [IL8]).* Red indicates a positive correlation and blue a negative correlation; each cell displays Pearson’s r. All listed correlations except PLAT met the false discovery rate threshold of 0.01; “ns” indicates a non-significant correlation at this threshold. These transcript-level correlations do not measure cell-surface ANXA2 protein or gene activity. Full gene names and exact results are provided in Supplementary Table S5.

In the TCGA-LIHC dataset, *ANXA2* expression demonstrated positive correlations with genes such as *F3, PLAU, PLAUR, SERPINE1, TNF, and CXCL8*, while it showed negative correlations with *FGA, FGB, FGG, VWF, PLG*, and *IL6R* (refer to Figure 4 and Table S5). Notably, *PLAUR* exhibited the strongest positive correlation (r = 0.479), whereas *IL6R* had the strongest negative correlation among the genes examined (r = −0.458). The correlation coefficients and adjusted *p*-values for all selected genes, including *PLAT*, are detailed in the table. It is crucial to recognize that these correlations within bulk-tissue transcripts do not indicate direct molecular or causal interactions.

### 2.10. Diagnostic Discrimination of ANXA2 Expression in TCGA-LIHC

In the TCGA-LIHC cohort, the mRNA expression of *ANXA2* in tissue effectively distinguished 371 primary HCC samples from 50 normal liver samples, with an area under the curve (AUC) of 0.895 (95% CI, 0.852–0.933; Figure S6). Utilizing Youden’s J statistic, a cutoff value of 5.327 was determined within the same cohort, resulting in a sensitivity of 76.5% and a specificity of 92.0%. However, these cutoff and performance metrics were not validated in an independent cohort.

## 3. Discussion

The main discovery is that *ANXA2* transcript expression was higher in HCC than in non-tumor liver tissue in TCGA-based analyses and the independent GSE25097 dataset. CPTAC data also showed higher *ANXA2* protein abundance in HCC. In TCGA-LIHC, higher *ANXA2* expression was associated with poorer overall survival. Correlations with immune-, coagulation-, fibrinolysis-, and inflammation-related genes describe a transcriptional pattern; they do not establish that *ANXA2* regulates these processes.

Research has consistently shown that *ANXA2* expression is elevated in HCC and correlates with decreased survival rates [3,22,23,24]. Despite these findings, neither the associations observed nor the current cross-sectional analysis provide conclusive evidence that *ANXA2* expression escalates as the tumor progresses or that it directly contributes to tumor aggressiveness. Notably, *ANXA2* expression levels were found to be higher in various stage and grade subgroups compared to normal liver tissue (Figure 1B,C). However, the small sample sizes of stage IV and grade 4 groups hinder comprehensive comparisons among tumor subgroups, and cross-sectional data alone cannot determine if expression levels increase during tumor progression.

The expression of *ANXA2* was correlated with a diverse transcriptional pattern that includes genes related to pericellular plasmin regulation, inflammation, and liver-derived hemostatic proteins. The observed positive and negative correlations do not suggest a consistent activation of coagulation or fibrinolysis. These findings warrant further investigation but do not establish *ANXA2* as a causal regulator of immunothrombotic processes.

The biological relevance of the positive correlations with *PLAU* and *PLAUR* lies in the role of the *uPA*–*uPAR* system, which governs pericellular proteolysis and is instrumental in processes such as extracellular matrix remodeling, cell migration, angiogenesis, and the metastatic spread of HCC and other solid tumors. Additionally, the ANXA2–S100A10 complex on the cell surface facilitates localized plasmin generation by binding to plasminogen and tissue-type plasminogen activator. Despite being molecularly distinct, both *ANXA2* and the *uPA*–*uPAR* system are involved in pericellular plasmin generation. Their correlated transcript expression in LIHC may reflect related proteolytic processes, but this analysis did not test whether they interact directly or act together. However, this study did not explore any direct molecular or functional interactions between these entities [25,26]. Moreover, the positive correlation between *ANXA2* and *SERPINE1*, which encodes plasminogen activator inhibitor-1, implies a concurrent regulation of both fibrinolytic-promoting and fibrinolytic-inhibitory components, rather than a simple increase in overall fibrinolytic activity [27].

The inverse relationships observed between *ANXA2* and *PLG*, the fibrinogen genes *FGA*, *FGB*, and *FGG*, and *VWF* warrant careful consideration. These correlations are based on mRNA levels in bulk tumor tissue, and thus, a decrease in *PLG* expression should not be interpreted as plasminogen consumption, which is a protein-level phenomenon. Instead, these inverse relationships might indicate variations in cellular composition, a decline in differentiated hepatocyte functions, or a decrease in liver synthetic programs in tumors with elevated *ANXA2* expression. This interpretation is supported by the negative enrichment of pathways associated with bile-acid biosynthesis, fatty-acid degradation, peroxisomal function, lipoprotein remodeling, and intrinsic fibrin-clot formation. Therefore, the findings suggest a transcriptional reorganization of hepatic metabolic and hemostatic programs linked to *ANXA2* expression, rather than a broad activation of coagulation [28].

The association of *ANXA2* expression with an inflammatory transcriptional environment is evidenced by its positive correlations with *CXCL8* and *TNF*. In HCC, *CXCL8* plays a role in neutrophil recruitment, angiogenesis, and tumor-cell invasion, while *TNF*-related signaling is involved in chronic inflammatory processes linked to hepatocarcinogenesis [29]. The stronger correlation coefficient for *CXCL8* suggests a potentially closer transcriptional link with *ANXA2* in this cohort. However, these correlations do not confirm that *ANXA2* directly regulates the expression of *CXCL8* or *TNF*, nor do they demonstrate the functional activity of these pathways.

The inverse relationship between *ANXA2* and *IL6R* is intriguing, particularly in light of the well-documented role of IL-6 signaling in the development and progression of HCC [30]. Despite this, correlations derived from bulk-tissue transcript data are insufficient to clarify whether this pattern is due to variations in cellular composition, decreased expression of membrane-bound *IL6R*, receptor shedding, changes in classical IL-6 signaling, or an increased reliance on IL-6 trans-signaling. Consequently, the current analysis does not elucidate the functional connection between *ANXA2* and *IL6R*. To resolve these uncertainties, it would be necessary to conduct cell-specific expression analyses and experiments that assess both classical and trans-signaling pathways.

*ANXA2* expression was found to be positively linked with several genes associated with immune checkpoints and macrophages, such as HAVCR2 and CSF1R. However, since these observations are derived from bulk tumor tissue, it is not possible to determine which specific cells are expressing these genes or to confirm the presence of checkpoint activity, increased macrophage infiltration, or macrophage polarization. These patterns might partly reflect variations in the proportions of tumor, immune, and stromal cells across different samples. In a study on another gene, *PAQR5*, related to HCC, Yang et al. utilized a combination of bulk, single-cell, and spatial transcriptomic analyses to investigate gene expression across different cell types and tissue locations [31]. A similar approach, focusing on cell-specific analysis and protein-level evaluation, could help identify where *ANXA2* and its associated immune markers are expressed in HCC tissue. The current data do not clarify whether *ANXA2* influences immune-cell function.

Gene set enrichment analysis provided a broader transcriptional context for the correlations linked to *ANXA2*. The analysis revealed positive enrichment in gene sets associated with phagocytosis and translation, while those related to liver metabolism and certain coagulation processes showed negative enrichment. Although a previous study on HCC also explored *ANXA2*-related signaling pathways, [3,22,23,24] the differences in analytical methods prevent direct comparison. Our findings highlight expression patterns but do not confirm that ANXA2 directly influences the activation or inhibition of these pathways.

GSE25097 corroborated the observation that *ANXA2* transcript expression is elevated in HCC compared to non-tumorous liver tissue; however, it did not confirm the results of the receiver operating characteristic (ROC) analysis. The ROC analysis conducted on the TCGA-LIHC cohort demonstrated the ability to distinguish between tumor and normal tissue samples, with an AUC of 0.895. The cutoff, sensitivity, and specificity were estimated and evaluated using the same sample set. Although published studies have investigated circulating *ANXA2* protein as a potential marker for HCC [32,33], their findings are not directly comparable to this tissue mRNA analysis due to differences in specimen types and comparison groups. Therefore, independent validation in clinically representative cohorts is necessary to ascertain the diagnostic efficacy of *ANXA2*.

This study acknowledges several limitations. Primarily, the analyses relied on retrospective bulk-tissue datasets, with multiple platforms incorporating overlapping TCGA samples. Consequently, the observed concordance in TCGA-based analyses indicates analytical agreement rather than independent replication. Bulk-tissue correlations are limited in identifying the cellular origin of *ANXA2* or its correlated genes and may be influenced by tumor purity and variations in the abundance of hepatocytes, immune cells, endothelial cells, and stromal cells. Additionally, correlation and enrichment analyses do not establish causality, direct molecular interactions, pathway activity, fibrinolytic function, or immune-cell states. The small number of Stage IV and Grade 4 tumors further constrains the interpretation of subgroup differences. Although GSE25097 externally validated the differential expression of *ANXA2*, the associations with survival, ROC performance, and the immune–hemostatic correlation pattern require validation in independent, clinically annotated cohorts. The datasets lacked paired tumor-tissue *ANXA2* mRNA and circulating *ANXA2* protein measurements from the same patients, so we could not assess whether tissue expression correlates with blood *ANXA2* concentrations. Cell-specific, spatial, protein-level, and functional studies are needed to evaluate the proposed relationships among *ANXA2*, pericellular fibrinolysis, coagulation-related genes, and immune remodeling.

## 4. Materials and Methods

### 4.1. Study Design and Data Sources

This research adopted a comprehensive bioinformatics approach, integrating transcriptomic, proteomic, clinical, survival, immune, and pathway-level analyses to explore *ANXA2* as a potential molecular hub in tumor-associated immunothrombotic remodeling within HCC. The analyses were conducted using publicly accessible, de-identified datasets. The primary focus of the transcriptomic analyses was the TCGA-LIHC cohort. Various analytical platforms were employed to investigate different facets of *ANXA2*-associated biology. For pan-cancer expression and immune infiltration analyses, TIMER was utilized [34], GEPIA2 (Gene Expression Profiling Interactive Analysis 2; version 2) facilitated tumor–normal expression comparisons and immune-related correlation analyses [35], while UALCAN (https://ualcan.path.uab.edu/, accessed on 15 March 2026) was used for clinicopathological and proteomic analyses [36]. Protein-level expression was further examined using data from HPA and CPTAC [36,37]. Prognostic associations were assessed through survival analyses, with external validation provided by the Kaplan–Meier Plotter [38]. LinkedOmics (https://www.linkedomics.org/, accessed on 30 March 2026) was employed for *ANXA2* co-expression, functional enrichment, and correlation analyses involving genes related to coagulation, fibrinolysis, and inflammation [39]. Given the overlap in TCGA-derived data across several analytical platforms, concordant findings were considered complementary analytical evidence rather than independent biological validation. Consequently, independent transcriptomic validation of *ANXA2* expression was conducted using the independent Gene Expression Omnibus dataset GSE2509 [40]. Supplementary Table S1 outlines the data sources, objectives, cohorts, and independence for each analysis. Analyses using TCGA-LIHC data through various portals were regarded as analyses of overlapping samples rather than as independent validation.

### 4.2. Pan-Cancer Expression of ANXA2 in LIHC

Pan-cancer expression analysis of *ANXA2* was performed using the (TIMER2.0; http://timer.cistrome.org/, accessed on 1 March 2026) platform [34] which facilitates the comparison of gene expression between tumor and normal tissues within TCGA cancer types. The analysis initially aimed to identify the general expression pattern of *ANXA2* across TCGA tumor types, with a specific focus on the TCGA-LIHC cohort. Expression levels in tumor and normal tissues were compared using the statistical methods provided by TIMER, with significance assessed based on the platform’s *p*-values. To further explore *ANXA2* dysregulation in HCC, the GEPIA tool [35] was employed. This tool integrates TCGA tumor data with TCGA and GTEx normal tissue data, allowing for a comparison of *ANXA2* expression in HCC versus non-tumor liver tissues through its default differential-expression analysis. This analysis served as additional evidence of *ANXA2* expression dysregulation, complementing the findings rather than acting as an independent validation cohort.

### 4.3. Association of ANXA2 Expression with Clinicopathological Features

The UALCAN web portal was employed to examine the correlation between *ANXA2* expression and clinicopathological characteristics within the TCGA-LIHC cohort [36]. Initially, *ANXA2* transcript levels were assessed by comparing normal liver tissues (*n* = 50) with primary HCC tissues (*n* = 371). Subsequently, expression levels were analyzed in relation to pathological stages, specifically Stage I (*n* = 168), Stage II (*n* = 84), Stage III (*n* = 82), and Stage IV (*n* = 6), as well as tumor grades, including Grade 1 (*n* = 54), Grade 2 (*n* = 173), Grade 3 (*n* = 118), and Grade 4 (*n* = 12). Statistical analyses were conducted using UALCAN’s tools, and the platform’s *p*-values were utilized to determine statistical significance. These analyses aimed to explore the association between *ANXA2* expression and clinicopathological indicators of HCC progression.

### 4.4. Protein Level Expression of ANXA2

Two complementary proteomic resources were used to assess ANXA2 protein expression in HCC. First, we used CPTAC protein abundance data accessed through the UALCAN portal to compare the levels of ANXA2 protein in primary HCC tissues (*n* = 165) and normal liver tissues (*n* = 165) [36]. We also investigated ANXA2 protein expression at the tissue level by utilizing the HPA and publicly available immunohistochemical staining images of HCC specimens [37]. Representative HPA images were evaluated for the presence and relative staining pattern of ANXA2 protein in HCC tissue. The CPTAC quantitative proteomic data and HPA immunohistochemical evidence were used in combination as complementary protein-level support for the transcriptomic findings.

### 4.5. Association of ANXA2 Expression with Overall Survival

The prognostic value of *ANXA2* expression in HCC was assessed with overall survival as the primary clinical endpoint. Kaplan–Meier survival analyses were conducted utilizing publicly accessible survival-analysis tools, such as the HPA and Kaplan–Meier Plotter [37,38]. Patients were categorized into high- and low-*ANXA2* expression groups based on the grouping criteria of the respective platforms. The differences in overall survival between these expression groups were analyzed using the log-rank test, and HRs with 95% CIs were documented when available from the platforms. Furthermore, patient-level TCGA-LIHC expression and clinical data were examined to determine whether *ANXA2* expression was associated with overall survival independently of major clinicopathological variables. After excluding cases with missing data for variables included in the model, 337 patients with complete survival, expression, and clinicopathological information were retained, including 114 observed death events. *ANXA2* expression and age were treated as continuous variables, sex was included as a binary variable, and pathological stage was classified as early stage (Stages I–II) or advanced stage (Stages III–IV). A multivariable Cox proportional hazards regression model, incorporating *ANXA2* expression, age, sex, and pathological stage, was fitted in Python (version 3.14.8) using the CoxPHFitter implementation of the lifelines package. Hazard ratios with corresponding 95% CIs and *p*-values were calculated.

### 4.6. Associations of ANXA2 with Immune-Related Features

The investigation into the link between *ANXA2* expression and immune-cell infiltration in HCC was conducted using the TIMER tool [34]. This study utilized the TCGA-LIHC cohort to analyze six key immune-cell types: B cells, CD8^+^ T cells, CD4^+^ T cells, macrophages, neutrophils, and dendritic cells. The correlation between *ANXA2* expression and the estimated abundance of these immune cells was assessed, with tumor purity considered as per TIMER’s analytical framework. Correlation coefficients and *p*-values provided by TIMER were employed to determine the strength and significance of these associations. Additionally, the TIMER survival module was used to explore the relationship between immune-cell infiltration and patient outcomes. Kaplan–Meier analyses were conducted to assess overall survival based on *ANXA2* expression and the estimated abundance of each immune-cell type. These analyses aimed to ascertain whether the prognostic significance of *ANXA2* in HCC was associated with variations in the tumor microenvironment’s immune composition.

### 4.7. Correlation of ANXA2 with Genes Associated with Immune Checkpoints and Macrophages

To explore the immunoregulatory environment linked to *ANXA2* expression in HCC, correlation analyses were conducted using GEPIA, drawing on TCGA-LIHC expression data [35]. The study focused on the relationships between *ANXA2* expression and specific immune checkpoint genes, such as *PDCD1 (PD-1), CD274 (PD-L1), CTLA4,* HAVCR2 *(TIM-3), TIGIT, LAG3, PDCD1LG2 (PD-L2),* and *IDO1*. Furthermore, the analysis included macrophage-associated markers like *CD68, CD163, MS4A4A*, and CSF1R to understand the connection between *ANXA2* expression and macrophage-related characteristics in the HCC tumor microenvironment. The correlation coefficients and *p*-values provided by GEPIA were utilized to evaluate the direction, strength, and statistical significance of these associations.

### 4.8. Functional Enrichment and Pathway Analysis

To explore the biological processes and pathways associated with *ANXA2* expression in HCC, a gene co-expression analysis was conducted using the LinkedOmics database within the TCGA-LIHC cohort [39]. Genes correlated with *ANXA2* expression were prioritized based on statistical outcomes from LinkedOmics. Subsequently, gene set enrichment analysis was performed using the LinkInterpreter module. Functional enrichment was evaluated through KEGG and Reactome pathway annotations. The enrichment results were assessed using normalized enrichment scores and false discovery rate (FDR), with an FDR threshold of less than 0.05 indicating statistical significance. Pathways enriched in immune signaling, inflammation, coagulation, and fibrinolysis were further analyzed to elucidate the potential biological context linking *ANXA2* with immune and hemostatic processes in HCC.

### 4.9. Correlation Analysis of Coagulation, Fibrinolysis, and Inflammation-Related Genes

In the TCGA-LIHC cohort, LinkedOmics was employed to perform correlation analyses aimed at exploring the connection between *ANXA2* and the molecular pathways involved in tumor-associated immunothrombotic remodeling [39]. A comprehensive analysis was conducted on a selected panel of genes involved in coagulation, fibrinolysis, and inflammatory signaling. The genes associated with coagulation included *F3, FGA, FGB, FGG,* and *VWF*. For fibrinolysis, the genes examined were *PLAU, PLAT, PLG, PLAUR*, and *SERPINE1*. The inflammation-related genes comprised IL8, *TNF*, and *IL6R*. To assess the direction and strength of the associations between *ANXA2* and each gene, Pearson correlation coefficients were calculated. The statistical significance of these associations was determined using *p*-values derived from the platform, with adjustments for multiple testing performed using the Benjamini–Hochberg FDR correction. This study aimed to elucidate the relationship between *ANXA2* and the molecular features related to coagulation, fibrinolysis, and inflammation in HCC.

### 4.10. External Transcriptomic Validation

External Transcriptomic Validation: To independently evaluate *ANXA2* expression in HCC, the external transcriptomic dataset GSE25097 was retrieved from the Gene Expression Omnibus (GEO) database [40]. This analysis involved a comparison of gene expression profiles between HCC tumor tissues and adjacent non-tumor liver tissues, utilizing a microarray-based transcriptomic platform. The assessment of differential expression was conducted using log_2_fold-change values and Benjamini–Hochberg-adjusted *p*-values. Genes with |log_2_fold change| ≥ 0.585 and an adjusted *p*-value < 0.01 were considered differentially expressed and were visualized in a volcano plot to represent the overall differential expression profile [41,42]. *ANXA2* was specifically analyzed as the gene of interest, with a focus on the direction and statistical significance of its differential expression between HCC and adjacent non-tumor tissues.

### 4.11. Receiver Operating Characteristic Analysis

The analysis of ROC curves was conducted using expression data from TCGA-LIHC to assess the efficacy of *ANXA2* expression in differentiating primary HCC tumor tissues from normal liver samples. The dataset included 371 primary tumor samples and 50 normal samples, categorized as tumor (1) or normal (0), with *ANXA2* expression as the continuous predictor. Python’s scikit-learn package facilitated the ROC analysis, and the AUC was calculated to evaluate discriminatory performance. The optimal cutoff for *ANXA2* expression was determined using Youden’s J statistic (sensitivity + specificity − 1). Sensitivity, specificity, and overall classification accuracy were computed at this threshold. The 95% CI for the AUC was estimated through 2000 bootstrap resamples, with the 2.5th and 97.5th percentiles of the bootstrap AUC distribution serving as the confidence limits. A fixed random seed of 42 ensured reproducibility. As the ROC analysis and cutoff optimization were conducted within the same TCGA-LIHC cohort, the results were interpreted as reflecting in-cohort discriminatory performance rather than independently validated diagnostic accuracy.

### 4.12. Statistics Analysis

Statistical analyses were conducted utilizing the procedures available in the respective bioinformatics platforms, as well as the additional statistical methods previously described. Unless otherwise indicated, statistical significance for TIMER, GEPIA, and UALCAN analyses was defined as *p* < 0.01. Multiple-testing-adjusted criteria, including the FDR, were applied as appropriate for enrichment analyses. Statistical analyses and data visualization were executed using R (version 4.5.1) and Python (version 3.14.8). All tests were two-sided where applicable.

## 5. Conclusions

In this study *ANXA2* was found to be consistently overexpressed at both the transcript and protein levels in HCC, with its elevated expression linked to a decrease in overall survival. Furthermore, *ANXA2* expression was associated with a complex transcriptional pattern that involved the *uPA–uPAR* system, tissue factor, inflammatory mediators, immune-regulatory markers, and a reduction in the expression of several liver-derived metabolic and hemostatic genes. These findings suggest that *ANXA2* could play a role in immune–hemostatic remodeling in HCC. However, before *ANXA2* can be confirmed as a prognostic biomarker or a causal molecular regulator of tumor-associated immunothrombosis, further studies are needed, including independent prognostic validation and cell-resolved, protein-level, and functional analyses.

## Figures and Tables

**Table 1:** Univariate and multivariable Cox proportional hazards regression analyses for overall survival in TCGA-LIHC.

Analysis	Covariate	HR	95% CI	*p*-Value
Univariate	*ANXA2* expression, per one-unit increase	1.29	1.09–1.53	**0.003**
Multivariable	*ANXA2* expression, per one-unit increase	1.26	1.07–1.49	**0.006**
Multivariable	Age, per year	1.01	1.00–1.03	0.147
Multivariable	Sex, male vs. female	0.89	0.61–1.31	0.558
Multivariable	Pathological stage, advanced vs. early	2.48	1.70–3.61	**<0.001**

Note: The analysis encompassed 337 patients diagnosed with liver hepatocellular carcinoma (LIHC) from the TCGA database, with 114 recorded deaths. *ANXA2* expression and age were treated as continuous variables, with hazard ratios (HRs) indicating the effect of a one-unit increase in log_2_-transformed expression and a one-year increment in age. Female sex and early pathological stages (I–II) served as reference categories, while stages III–IV were classified as advanced. The multivariable model, which included *ANXA2* expression, age, sex, and pathological stage, achieved a concordance index of 0.64. Abbreviations: CI, confidence interval; HR, hazard ratio; LIHC, liver hepatocellular carcinoma. Bold values indicate statistical significance (*p* < 0.05).

## Data Availability

The datasets examined in this research are accessible to the public. Transcriptomic and clinical data for TCGA-LIHC were retrieved from The Cancer Genome Atlas via the Genomic Data Commons and its analytical tools. Proteomic and immunohistochemical data were sourced from the Clinical Proteomic Tumor Analysis Consortium and the Human Protein Atlas. Validation of external transcriptomic data was achieved using the Gene Expression Omnibus dataset GSE25097. All processed data that underpin the study’s conclusions are included in the article and its Supplementary Materials, and additional analysis files are available from the corresponding author upon reasonable request.

## Supplementary Materials

The following supporting information can be downloaded at https://www.mdpi.com/article/10.3390/ijms27198915/s1.
