# Supplementary material for: ANXA2 as a Candidate Molecular Hub Linking Fibrinolysis, Coagulation, and Immune Remodeling in Hepatocellular Carcinoma: An Integrative Multi-Omics Analysis
Source: Int J Mol Sci. 2026 Oct 7;27(19):8915. doi: 10.3390/ijms27198915 (PMC13666242; doi:10.3390/ijms27198915)
Supplement: Supplementary file 1 [file ijms-27-08915-s001.zip › ijms-4571139-supplementary.pdf]

Supplementary Materials

Table S1. Overview of data sources and analytical workflow.

Data source or platform	Purpose in this study	Cohort or material analyzed	Independent validation?
TCGA-LIHC, accessed through TIMER	Pan-cancer and HCC expression comparisons; estimated immune-cell associations	TCGA tumor and available normal tissue data; TCGA-LIHC for HCC analyses	No; overlaps with other TCGA-based analyses
TCGA-LIHC and GTEx, accessed through GEPIA	Tumor–normal expression comparison and correlations with immune-related genes	TCGA-LIHC tumors and normal liver samples drawn from TCGA and GTEx, as applicable to the analysis	No; the tumor data overlap with other TCGA-based analyses
TCGA-LIHC, accessed through UALCAN	ANXA2 expression by tumor status, pathological stage, and grade	371 primary HCC and 50 normal samples for the reported tumor–normal comparison; subgroup sizes are given in Section 2.3	No; TCGA-based analysis
CPTAC, accessed through UALCAN	Quantitative comparison of ANXA2 protein abundance	165 HCC and 165 normal liver samples, as reported in Section 2.4	Separate protein dataset; not the external transcriptomic validation cohort
Human Protein Atlas (HPA)	Examination of representative ANXA2 immunohistochemical staining and a survival analysis	Public HCC tissue images and the cohort used by the HPA survival tool	No independent validation claim is made
TCGA-LIHC patient-level data	Multivariable overall-survival model and tumor–normal ROC analysis	337 complete cases for the Cox model; 371 tumors and 50 normal samples for ROC analysis	No; both are TCGA-based analyses
Kaplan–Meier Plotter	Additional overall-survival analysis	HCC cohort available through the platform	Cohort overlap should be checked before calling this independent validation
TCGA-LIHC, accessed through LinkedOmics	Co-expression, pathway enrichment, and correlations with hemostatic and inflammatory genes	TCGA-LIHC transcriptomic data	No; TCGA-based analysis
GEO dataset GSE25097	External assessment of ANXA2 differential expression in HCC	HCC tumors and adjacent non-tumor liver tissues	**Yes, for ANXA2 differential expression only**

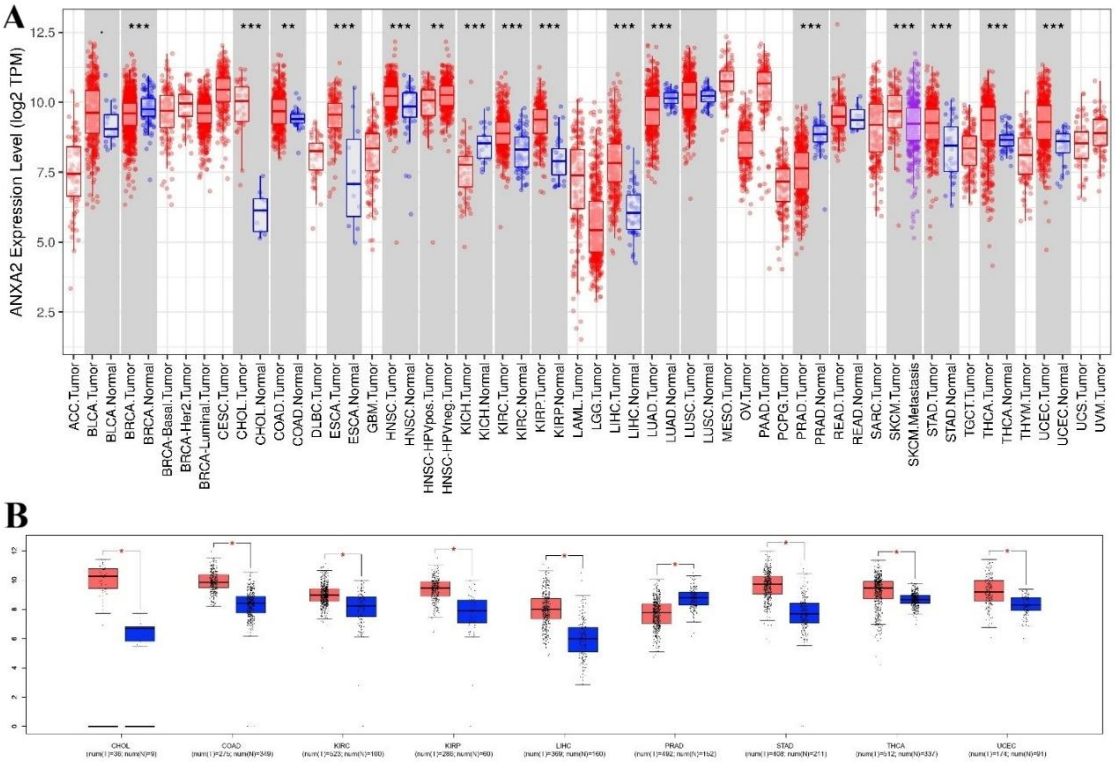

Figure S1. Pan-cancer expression analysis of ANXA2 using TIMER and GEPIA. (A) TIMER was employed to evaluate ANXA2 expression across various TCGA cancer types. The boxplots depict a comparison between primary tumor tissues (red) and their corresponding normal tissues (blue), with expression levels shown as log_2-transformed transcripts per million (TPM). Each data point signifies an individual sample. ANXA2 expression was notably higher in liver hepatocellular carcinoma (LIHC) tissues compared to normal liver tissues. (B) GEPIA was used to analyze ANXA2 expression across selected cancer types, integrating data from TCGA tumor and TCGA/GTEx normal tissues. Expression levels are presented as $log_2(TPM + 1)$, with tumor and normal tissues represented in red and blue, respectively. Consistent with the TIMER findings, ANXA2 expression was significantly elevated in LIHC tumor tissues compared to normal liver tissues. Statistical significance was assessed using the platform-specific methods detailed in the Methods section, with significance levels indicated as follows: $p < 0.01$ (*), $p < 0.001$ (**), $p < 0.0001$ (***).

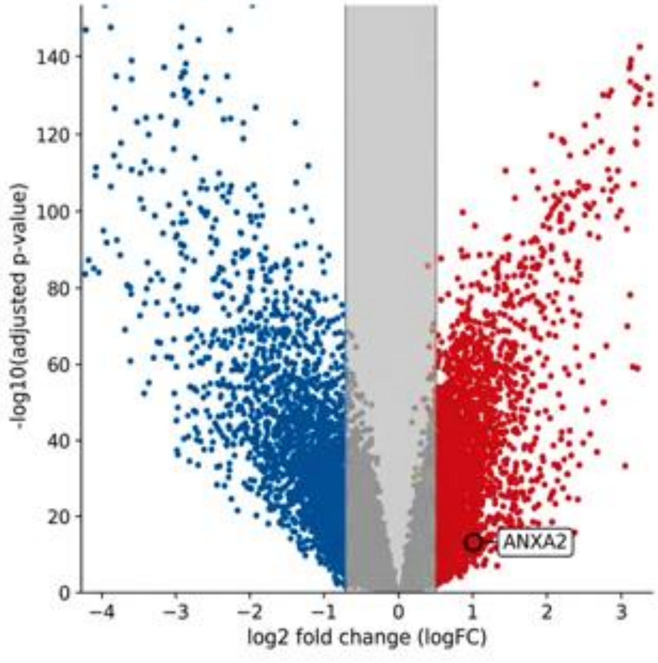

Figure S2. ANXA2 expression in GSE25097 is independently validated. The volcano plot visualizes the differential gene expression between hepatocellular carcinoma (HCC) tissues (n = 268) and adjacent non-tumor liver tissues (n = 243) in this cohort. On the x-axis, the $\log_2$ fold change ($\log_2$FC) is shown, representing the expression level in HCC compared to adjacent non-tumor tissue. The y-axis displays the $-\log_{10}$(adjusted p-value). Genes are considered differentially expressed if they meet the criteria of $|\log_2 FC| \geq 0.585$ and an adjusted p-value < 0.01. Genes with significantly increased expression are marked in red, while those with decreased expression are in blue; genes not meeting these criteria are shown in grey. ANXA2, indicated by a black circle, shows a marked increase in expression in HCC tissues.

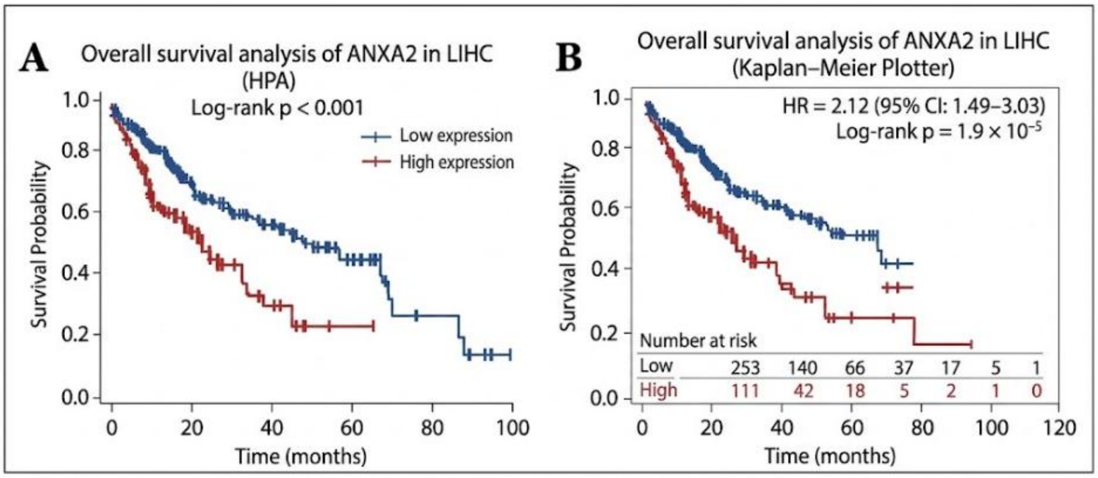

Figure S3. Complementary survival analyses of ANXA2 expression in LIHC.(A) Kaplan–Meier overall survival curves obtained from the Human Protein Atlas, showing significantly poorer survival among patients with high ANXA2 expression (pink) than among those with low expression (blue; log-rank $p < 0.001$). (B) Overall survival analysis obtained using Kaplan–Meier Plotter. Patients with high ANXA2 expression (red; n = 111) had poorer overall survival than those with low expression

(blue; n = 253; HR = 2.12, 95% CI: 1.49–3.03; log-rank $p = 1.9 \times 10^{-5}$). Vertical marks indicate censored observations, and the number-at-risk table is shown for the Kaplan–Meier Plotter analysis. Expression-group cutoffs were determined using the platform-specific procedures described in the Methods. Abbreviations: CI, confidence interval; HR, hazard ratio; LIHC, liver hepatocellular carcinoma.

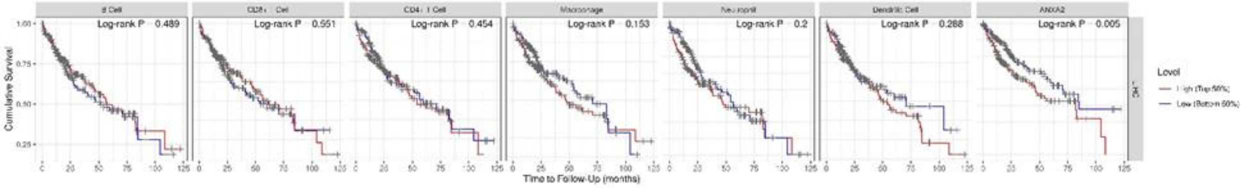

Figure S4. Associations of estimated immune-cell abundance and ANXA2 expression with overall survival in LIHC. Kaplan–Meier survival curves, generated using TIMER, compare patients with high (top 50%; red) and low (bottom 50%; blue) estimated levels of B cells, $CD8^+$ T cells, $CD4^+$ T cells, macrophages, neutrophils, and dendritic cells. The analysis indicates that none of the estimated immune-cell abundances was significantly associated with overall surviva: B cells (p = 0.489), $CD8^+$ T cells (p = 0.551), $CD4^+$ T cells (p = 0.454), macrophages (p = 0.153), neutrophils (p = 0.200), and dendritic cells (p = 0.288). Conversely, elevated ANXA2 expression correlates with reduced overall survival (log-rank p = 0.005). Vertical marks denote censored data points.

Table S2. Correlations between ANXA2 expression and immune-checkpoint and macrophage-associated genes in LIHC

**Immune-checkpoint genes**			
**Gene**	**Common protein name**	**R**	**p-value**
PDCD1	PD-1	0.29	**1.1×10^{-8}**
CD274	PD-L1	0.18	**5.9×10^{-4}**
CTLA4	CTLA-4	0.32	**5.9×10^{-10}**
HAVCR2	TIM-3	0.45	**1.0×10^{-19}**
TIGIT	TIGIT	0.28	**5.4×10^{-8}**
LAG3	LAG-3	0.016	0.76
PDCD1LG2	PD-L2	0.17	**0.0014**
IDO1	IDO1	0.12	0.026
**Macrophage-associated genes**			
**Gene**	**General association**	**R**	**p-value**
CD68	Pan-macrophage marker	0.37	**2.5×10^{-13}**
CD163	Macrophage/M2-like marker	0.21	**4.0×10^{-5}**
MS4A4A	Macrophage/M2-like marker	0.34	**1.3×10^{-11}**

CSF1R	Macrophage-lineage marker	0.45	**1.4×10^{-19}**

Note; R represents the correlation coefficient. Statistical significance was defined as $p < 0.01$.

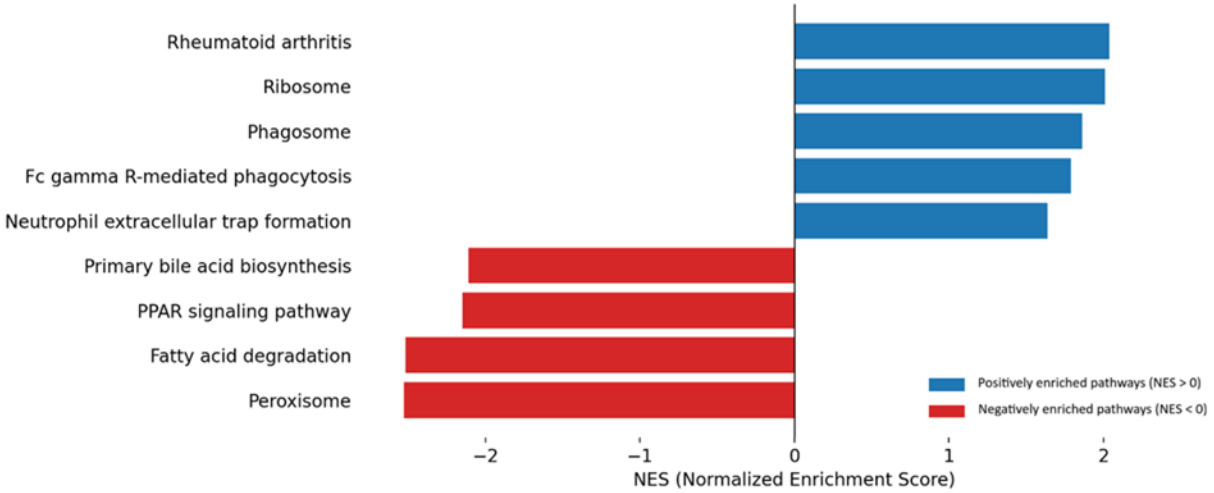

Figure S5. KEGG gene set enrichment analysis of the ANXA2-associated transcriptional profile in LIHC. This analysis was conducted using LinkedOmics, with genes ranked by their correlation with ANXA2 expression within the TCGA-LIHC cohort. The figure displays selected KEGG pathways that are significantly enriched, as determined by their normalized enrichment scores (NES). Pathways positively enriched among genes with elevated ANXA2 expression are represented by blue bars, while red bars denote negatively enriched pathways. It is important to note that positive and negative enrichment reflect the direction of association with the ANXA2-correlated gene profile and do not imply pathway activation or inhibition. The statistical significance of the results was evaluated using the false discovery rate (FDR).

Table S3. KEGG gene set enrichment analysis of the ANXA2-associated transcriptional profile in TCGA-LIHC.

Enrichment direction	KEGG pathway	NES	FDR	Functional theme
Positive	Rheumatoid arthritis	2.04	$<2.2 \times 10^{-16}$	Inflammatory and immune signaling
Positive	Ribosome	2.01	$<2.2 \times 10^{-16}$	Ribosomal and translational activity
Positive	Phagosome	1.87	0.0021	Phagocytic activity
Positive	Fc gamma R-mediated phagocytosis	1.79	0.0099	Fc receptor-mediated phagocytosis
Positive	Neutrophil extracellular trap formation	1.65	0.026	Neutrophil and NET-associated processes
Negative	Peroxisome	−2.53	$<2.2 \times 10^{-16}$	Peroxisomal metabolism

Negative	Fatty acid degradation	−2.53	$<2.2 \times 10^{-16}$	Fatty-acid catabolism
Negative	PPAR signaling pathway	−2.15	$<2.2 \times 10^{-16}$	Lipid and metabolic regulation
Negative	Primary bile acid biosynthesis	−2.11	$<2.2 \times 10^{-16}$	Liver-associated bile-acid metabolism

Note; Genes were ranked according to their association with ANXA2 expression in the TCGA-LIHC cohort. Positive and negative NES values indicate enrichment toward the positively and negatively correlated ends of the ranked gene list, respectively. They do not indicate direct pathway activation or inhibition by ANXA2. Size represents the number of pathway genes included in the analysis. Abbreviations: FDR;false discovery rate, NES; normalized enrichment score.

Table S4. Reactome gene set enrichment analysis of the ANXA2-associated transcriptional profile in TCGA-LIHC.

**Enrichment direction**	**Reactome pathway**	**NES**	**FDR**	**Functional theme**
Positive	Formation of a pool of free 40S subunits	2.07	$<2.2 \times 10^{-16}$	Ribosomal-subunit availability
Positive	Peptide chain elongation	2.07	$<2.2 \times 10^{-16}$	Protein translation
Positive	Eukaryotic translation initiation	2.05	$<2.2 \times 10^{-16}$	Translation initiation
Positive	SRP-dependent cotranslational protein targeting to membrane	2.03	$<2.2 \times 10^{-16}$	Membrane and secretory-protein processing
Negative	Intrinsic pathway of fibrin clot formation	−2.18	$<2.2 \times 10^{-16}$	Coagulation and fibrin formation
Negative	Plasma lipoprotein remodeling	−2.22	$<2.2 \times 10^{-16}$	Lipoprotein metabolism
Negative	Phase I—functionalization of compounds	−2.23	$<2.2 \times 10^{-16}$	Hepatic xenobiotic metabolism
Negative	Peroxisomal protein import	−2.31	$<2.2 \times 10^{-16}$	Peroxisomal function
Negative	Peroxisomal lipid metabolism	−2.34	$<2.2 \times 10^{-16}$	Lipid metabolism

Note; Genes were ranked according to their association with ANXA2 expression in the TCGA-LIHC cohort. Positive and negative NES values indicate enrichment toward the positively and negatively correlated ends of the ranked gene list, respectively. They do not indicate direct pathway activation or inhibition by ANXA2. Size represents the number of pathway genes included in the analysis. Abbreviations: FDR; false discovery rate, NES; normalized enrichment score.

Table S5. Pearson correlations between ANXA2 expression and coagulation-, fibrinolysis-, and inflammation-related genes in TCGA-LIHC.

Category	Gene	Full gene name	Pearson's r	*p*-value	BH-adjusted FDR
Coagulation	*F3*	Coagulation factor III, tissue factor	0.278	5.49×10^{-8}	3.53×10^{-7}
Coagulation	*FGA*	Fibrinogen alpha chain	−0.186	3.19×10^{-4}	9.17×10^{-4}
Coagulation	*FGB*	Fibrinogen beta chain	−0.157	2.38×10^{-3}	5.58×10^{-3}
Coagulation	*FGG*	Fibrinogen gamma chain	−0.162	1.72×10^{-3}	4.19×10^{-3}
Coagulation	*VWF*	Von Willebrand factor	−0.239	3.37×10^{-6}	1.49×10^{-5}
Fibrinolysis	*PLAU*	Plasminogen activator, urokinase	0.37	1.68×10^{-13}	3.39×10^{-12}
Fibrinolysis	*PLAT*	Plasminogen activator, tissue type	0.133	1.01×10^{-2}	2.03×10^{-2}
Fibrinolysis	*PLG*	Plasminogen	−0.417	4.42×10^{-17}	2.01×10^{-15}
Fibrinolysis	*PLAUR*	Plasminogen activator, urokinase receptor	0.479	1.20×10^{-22}	1.96×10^{-20}
Fibrinolysis	*SERPINE1*	Serpin family E member 1 (plasminogen activator inhibitor 1)	0.172	8.69×10^{-4}	2.26×10^{-3}
Inflammation	*IL6R*	Interleukin 6 receptor	−0.458	1.14×10^{-20}	1.13×10^{-18}
Inflammation	*TNF*	Tumor necrosis factor	0.219	2.00×10^{-5}	7.44×10^{-5}
Inflammation	*CXCL8* (IL8)	C-X-C motif chemokine ligand 8 (interleukin 8)	0.377	5.91×10^{-14}	1.32×10^{-12}

Note: Pearson's r indicates the direction and strength of the correlation between ANXA2 expression and each listed gene in TCGA-LIHC. Positive values indicate that expression levels tend to increase together; negative values indicate an inverse association. BH-adjusted FDR denotes the false discovery rate adjusted using the Benjamini–Hochberg procedure. At an adjusted FDR threshold of 0.01, all listed correlations except PLAT meet the threshold. Gene symbols are shown using their official nomenclature.

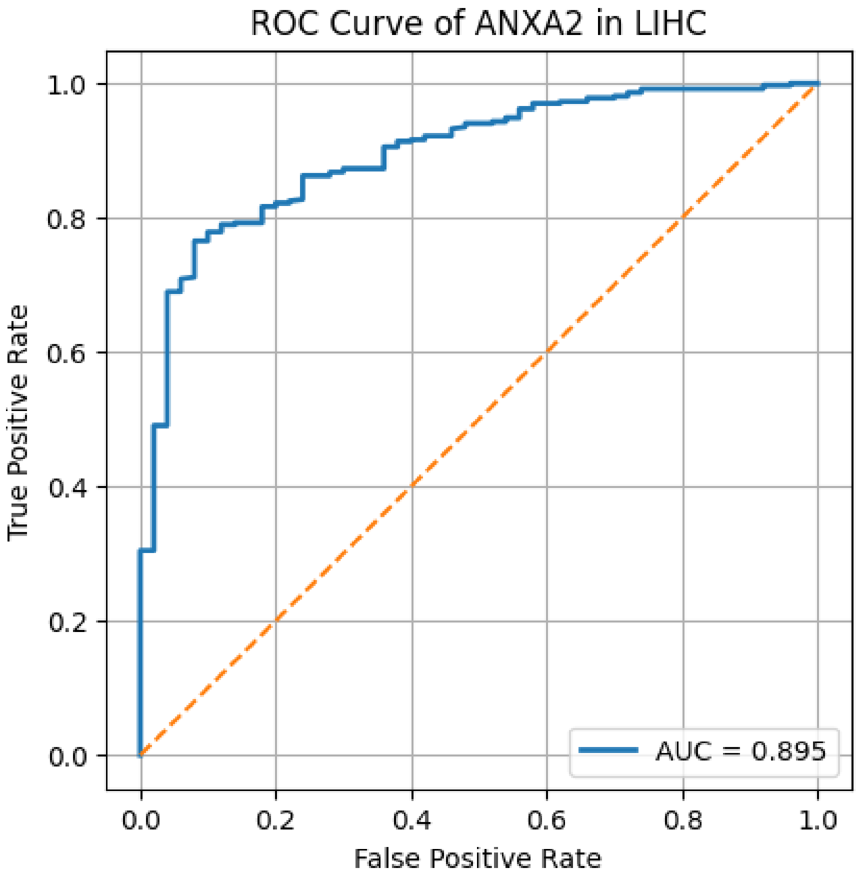

Figure S6. Evaluation of ANXA2's Discriminatory Capability within the TCGA-LIHC Cohort. The receiver operating characteristic (ROC) curve was employed to assess the efficacy of ANXA2 expression in differentiating primary hepatocellular carcinoma (HCC) tissues (n = 371) from solid-tissue normal liver samples (n = 50) within the TCGA-LIHC cohort. The area under the curve was determined to be 0.895 (95% CI: 0.852–0.933). A cutoff value of 5.327 in $\log_2$-transformed expression units was selected using Youden's J statistic, resulting in a sensitivity of 76.5% and a specificity of 92.0%. The diagonal line indicates the level of discrimination expected by chance. Performance estimates were both derived and evaluated within the same cohort.
